# Forearm vascular responses to mental stress in healthy older adults

**DOI:** 10.1002/phy2.180

**Published:** 2013-12-05

**Authors:** Matthew J. Heffernan, Hardikkumar M. Patel, Matthew D. Muller

**Affiliations:** 1 Pennsylvania State University College of Medicine, Penn State Hershey Heart and Vascular Institute, 500 University Drive, Hershey, 17033, Pennsylvania

**Keywords:** Blood pressure, psychological stress, skin blood flow, sympathetic nervous system, vasodilation

## Abstract

Forearm vascular conductance (FVC) increases in response to mental stress (verbal mental arithmetic) in young people. However, the effect of healthy aging and mental stress on FVC is unknown. In this study, we tested the hypothesis that FVC and cutaneous vascular conductance (CVC) would be attenuated in older adults compared to young adults. In 13 young (27 ± 1 year) and 11 older (62 ± 1 year) subjects, we quantified heart rate (HR), mean arterial pressure (MAP), FVC (Doppler ultrasound), and CVC (laser Doppler flowmetry) in response to a 3‐min bout of mental stress in the supine posture. Changes from baseline were compared between groups and physiological variables were also correlated. Older adults had a blunted HR response to mental stress (Δ = 7 ± 2 vs. 14 ± 2 beats/min) but ΔMAP was comparable between groups (Δ = 11 ± 2 mmHg vs. 9 ± 1). During the third minute of mental stress, the %ΔFVC (−2 ± 5 vs. 31 ± 12%) and %ΔCVC (2 ± 6 vs. 31 ± 15%) were both impaired in older adults compared to young subjects. There was no relationship between ΔHR and %ΔCVC in either group, but there was a positive relationship between ΔHR and %ΔFVC in both young subjects (*R* = 0.610, *P* < 0.027) and older subjects (*R* = 0.615, *P* < 0.044), such that larger tachycardia was associated with higher forearm vasodilation. These data indicate that older adults have impaired forearm vasodilation in response to mental stress.

## Abstract (precis)

Forearm vascular conductance (FVC) increases in response to mental stress (verbal mental arithmetic) in young people. However, the effect of healthy aging and mental stress on FVC is unknown. The current data indicate that older adults have impaired forearm vasodilation in response to mental stress due in part to an attenuated heart rate response.

## Introduction

Acute psychological stressors such as personal conflict, natural disasters, and bereavement can trigger adverse cardiovascular events in susceptible populations (Muller et al. 1994; Dimsdale 2008; Bei et al. 2013). These types of mental stress may be particularly detrimental to elderly individuals who have impaired vascular function, elevated ventricular stiffness, and heightened resting sympathetic tone compared with young people (Seals and Esler 2000; Monahan 2007; Prasad et al. 2007). However, the effect of healthy aging and mental stress on acute cardiovascular responses is not completely known. By studying how mental stress affects the cardiovascular system, we may gain insights into potential ways to attenuate its negative effects.

Bouts of mental stress (i.e., verbal fast‐paced mental arithmetic) activate the sympathetic nervous (SNS) (Delius et al. 1972; Muller et al. 2013d). Specifically, heart rate (HR), mean arterial pressure (MAP), and skin sympathetic nerve activity increase when the body is challenged with mental stress in the laboratory; muscle sympathetic nerve responses are variable (Rozanski et al. 1988; Yeung et al. 1991; Carter and Ray 2009; Durocher et al. 2011; Muller et al. 2013c). These acute hemodynamic changes serve to perfuse oxygenated blood throughout the body. Specifically, forearm vascular conductance (FVC), reflective of blood flow predominantly to the forearm skeletal muscle, increases during mental stress in young people (Dietz et al. 1994; Lindqvist et al. 1996; Halliwill et al. 1997; Carter et al. 2005b), but the effect of aging is unknown. Regarding forearm skin blood flow, older adults have impaired cutaneous vascular conductance (CVC) in response to whole body heating (i.e., a thermal activation of the SNS) (Kenney 1988; Holowatz et al. 2010), but the effect of mental stress (i.e., a cognitive activation of the SNS) on CVC is unknown. Therefore, the purpose of this investigation was to determine the effect of aging on hemodynamic and forearm vascular responses to mental stress. By using Doppler ultrasound and laser Doppler flowmetry, we tested the hypothesis that reflex increases in FVC and CVC to mental stress would be attenuated in healthy older adults.

## Material and Methods

### Subjects

All study protocols were approved in advance by the Institutional Review Board of the Penn State Milton S. Hershey Medical Center and conformed to the Declaration of Helsinki. A total of 13 young (7 women, 27 ± 1 year) and 11 older (6 women, 62 ± 1 year) subjects volunteered to participate and provided written informed consent. This sample size was determined based on the FVC response to mental stress (3 min average) between groups having greater than 80% power. Prior reports have shown no effect of gender on forearm vascular responses to mental stress so both men and women were enrolled (Ng et al. 1994; Yang et al. 2013). We did not specifically control for the menstrual cycle or use of oral contraceptives in the young women. All older women were postmenopausal and none were undergoing hormone replacement therapy. All subjects had supine resting blood pressures (BPs) below 125/80 mmHg and were nonasthmatic, nonobese, nonsmokers, not taking any prescription or vasoactive medication, and were in good health as determined by history and physical examination. All subjects reported being physically active, but none were competitive athletes. All older subjects underwent a Bruce treadmill protocol with 12 lead electrocardiogram (EKG) monitoring which was read by a cardiologist to rule out heart disease. Subjects refrained from caffeine, alcohol, and exercise for 24 h before the study and arrived to the laboratory in a semi‐fasted state (i.e., 4–6 h after their last meal).

### Instrumentation

All experiments were conducted in a dimly lit thermoneutral laboratory (22–25°C). Subjects were supine and clothed in a high‐density tube‐lined suit (Med‐Eng Systems, Ottawa, ON, Canada) that covered the entire body except for the feet, hands, both forearms, and head. Neutral water (34–35°C) was perfused through the suit to maintain mean skin temperature at a constant level and ensure that the obtained responses were due to the mental stress stimulus and not confounding effects of ambient temperature. Upon arrival to the laboratory, subjects were outfitted with a 3‐lead EKG (Cardiocap/5; GE Healthcare, Waukesha, WI) to monitor HR, a finger BP cuff (Finometer, FMS, Arnhem, The Netherlands), and a pneumotrace to monitor respiratory movement. The Finometer allows for the measurement of systolic blood pressure (SBP), diastolic blood pressure (DBP), and MAP on a beat‐by‐beat basis. We did not attempt to measure skin sympathetic nerve activity because (unlike thermal perturbations) it remains unclear if a given level of sympathetic outflow has an effect on skin blood flow under thermoneutral conditions and it also remains unclear how this translates into forearm blood flow (FBF).

The temporal resolution of Doppler ultrasound allows for the measurement of arterial blood flow on a beat‐by‐beat basis, whereas venous occlusion plethysmography (VOP) can only record 5–10 cycles per minute and includes both skin and muscle blood flow. Therefore, we used Doppler ultrasound (HDI 5000; ATL, Bothell, WA) to record brachial blood flow velocity in the left arm as previously described (Wilson et al. 2007; Muller et al. 2013a). Briefly, a 5–12 MHz linear transducer was placed over the brachial artery and the insonation angle was less than 60°. Brachial artery mean blood flow velocity was acquired in pulsed Doppler mode and velocity waveforms were synchronized to a data acquisition system (PowerLab; ADInstrument, New Castle, Australia) by a Doppler audio transformer (Herr et al. 2010). Cutaneous blood flow was measured by three laser Doppler probes placed on the right ventral forearm. The local temperature of the forearm skin measurement sites was maintained at 34°C by a local heater which also held the laser in place (Moor Instruments, Wilmington, DE). Prior to the protocol, resting BPs were obtained in triplicate by automated oscillometry of the left brachial artery (Philips Sure Signs VS3, Andover, MA) after 15 min of quiet rest. The average baseline brachial artery pressures (SBP, DBP, and MAP) were used to adjust the Finometer values during offline analysis. For example, if brachial MAP was 90 mmHg at baseline and the Finometer value for MAP was 85 mmHg, then 5 mmHg was added to all Finometer values in subsequent minutes. By doing this, we ensured that FVC was calculated with the true MAP value.

### Mental stress protocol

Following a 3‐min baseline period, mental stress occurred for 3 min and was followed by a 2‐min recovery period. Consistent with prior experiments, subjects were presented a two or three digit number and were asked to repeatedly subtract seven from each number (Yeung et al. 1991; Carter and Ray 2009; Klein et al. 2010; Ray and Carter 2010; Muller et al. 2013d). New numbers were given every 5–10 sec. Investigators continually encouraged the subject to verbally respond as fast as possible. Subjects were also told keep their eyes closed and their body relaxed. HR, MAP, and cutaneous blood flow flux were recorded continuously. Brachial blood flow velocity was recorded throughout the protocol except for 10–15 sec each minute when brachial artery diameters were obtained. Verbal and written time stamps were used to match hemodynamic and Doppler parameters during off‐line analysis. Perception of stress (0 = not stressful, 1 = somewhat stressful, 2 = stressful, 3 = very stressful, and 4 = very very stressful) was quantified after the bout of mental arithmetic (Callister et al. 1992).

### Data collection and statistical analysis

Forearm blood flow was calculated by multiplying the cross‐sectional area (*πr*^2^) of the vessel by mean brachial blood flow velocity and by 60 to express FBF in units of cm/sec. FVC was calculated as FBF/MAP and expressed as a percent change from baseline, consistent with prior reports (Carter et al. 2005b; Muller et al. 2013a). The three forearm skin blood flow sites were averaged and CVC was calculated as skin blood flow flux/MAP and then expressed as a percent change from baseline. All data are presented as averages of the last 15 sec of each minute. Presenting each minute of mental stress separately has been used in many prior publications (Carter et al. 2005b; Liu et al. 2006; Kuipers et al. 2008; Klein et al. 2010; Ray and Carter 2010; Schwartz et al. 2011; Yang et al. 2012; Muller et al. 2013c,d), but other studies have combined all minutes together (Yeung et al. 1991; Ng et al. 1994; Halliwill et al. 1997; Carter et al. 2002, 2005a; Carter and Ray 2009; Durocher et al. 2009; Pike et al. 2009; Yang et al. 2013). Therefore, we present the data in both ways.

Based on the study design, 2 group (young, older) by 4 time point (baseline, 1 min, 2 min, 3 min of mental stress) repeated measures analysis of variances (ANOVAs) were conducted using the absolute physiological units (SBP, DBP, MAP, HR, FBF, and FVC) in response to mental stress. Independent *t*‐tests were used to compare changes in physiological variables between age groups. Recovery from mental stress (minute 1 and minute 2) was also compared between groups. Based on previous studies (Pike et al. 2009; Yang et al. 2013), exploratory bivariate correlations between HR, MAP, FVC, and CVC were also conducted for the average responses (minutes 1, 2, and 3). Absolute physiological variables, absolute changes (Δ) from baseline, and percent changes from baseline (%Δ) were all considered for analysis, consistent with prior reports (Freyschuss et al. 1988; Ng et al. 1994; Carter and Ray 2009; Yang et al. 2013). Significance was set at *P* < 0.05 and data are presented as Mean ± SEM throughout.

## Results

Baseline parameters are displayed in Table 1. Resting BP was higher in the older adults, but resting HR, FBF, FVC, and CVC were comparable between groups. Preliminary analysis of sex differences in the young subjects revealed no group differences in physiological responses to mental stress so men and women were combined. Indeed, the ΔMAP (women: 10 ± 2 mmHg, men: 9 ± 2 mmHg, *P* = 0.659), ΔHR (women: 13 ± 1 beats/min, men: 16 ± 4 beats/min, *P* = 0.507), ΔFBF (women: 12 ± 3 mL/min, men: 19 ± 7 mL/min, *P* = 0.345), and ΔFVC (women: 40 ± 17%, men: 41 ± 23%, *P* = 0.990) were quite comparable in these young men and women.

**Table 1.** Resting baseline parameters prior to mental stress.

	Young	Older	*P*‐value
Age (years)	27 ± 1	62 ± 1	<0.001
Height (m)	1.74 ± 0.03	1.70 ± 0.04	0.368
Weight (kg)	74.0 ± 3.3	75.3 ± 2.7	0.576
BMI (kg/m^2^)	24.0 ± 0.1	25.9 ± 1.1	0.197
SBP (mmHg)	107 ± 1	121 ± 1	<0.001
DBP (mmHg)	64 ± 1	75 ± 2	<0.001
MAP (mmHg)	79 ± 1	88 ± 2	<0.001
HR (beats/min)	61 ± 2	60 ± 2	0.562
FBF (mL/min)	31 ± 5	33 ± 3	0.672
FVC (au)	0.40 ± 0.6	0.38 ± 0.05	0.887
Skin flux (au)	70 ± 11	92 ± 17	0.234
CVC (au)	0.89 ± 0.14	1.04 ± 0.18	0.516

Thirteen young subjects and 11 older subjects participated in this study and were measured in the supine posture. Blood pressure was obtained in triplicate by automated oscillometry after 15 min of quiet rest. Data are Mean ± SEM. BMI, body mass index; SBP, systolic blood pressure; DBP, diastolic blood pressure; MAP, mean arterial blood pressure; HR, heart rate; FBF, forearm blood flow; FVC, forearm vascular conductance; CVC, cutaneous vascular conductance; au, arbitrary units.

Perceived stress level in response to mental stress was 2.1 ± 0.2 in the young subjects and 2.1 ± 0.3 in the older subjects (*P* = 0.990). For the variables SBP, DBP, and MAP, repeated measures ANOVAs revealed a main effect for group (all *P* < 0.001) and a main effect for time (all *P* < 0.001), but no group by time interaction (all *P* > 0.4) such that the pressor response to mental stress was comparable between groups at all time points (Table 2). HR demonstrated a main effect for group (*P* = 0.025), a main effect for time (*P* < 0.001), and a group by time interaction (*P* = 0.038) such that older subjects had a blunted tachycardia in response to mental stress (Table 2).

**Table 2.** Hemodynamic and forearm vascular responses to mental stress

		Δ Minute 1	Δ Minute 2	Δ Minute 3	Average Δ	Δ Rec 1	Δ Rec 2
SBP							
Young		7 ± 2	13 ± 2	13 ± 3	11 ± 2	0 ± 2	0 ± 3
Older		11 ± 3	15 ± 2	14 ± 3	13 ± 2	2 ± 2	1 ± 3
DBP							
Young		6 ± 1	9 ± 1	8 ± 1	8 ± 1	0 ± 1	0 ± 1
Older		8 ± 1	9 ± 2	8 ± 3	9 ± 2	1 ± 2	0 ± 3
MAP							
Young		7 ± 1	11 ± 1	10 ± 1	9 ± 1	1 ± 1	0 ± 1
Older		9 ± 2	13 ± 2	11 ± 3	11 ± 2	2 ± 2	1 ± 2
HR							
Young		17 ± 3	13 ± 3	12 ± 1	14 ± 2	−5 ± 2	−4 ± 2
Older		*8 ± 1	*6 ± 2	*5 ± 2	*7 ± 2	−3 ± 1	−3 ± 2
FBF							
Young		20 ± 7	14 ± 3	12 ± 3	15 ± 4	7 ± 4	6 ± 4
Older		7 ± 3	*3 ± 2	*2 ± 2	*4 ± 2	*−4 ± 4	*−5 ± 3
FVC							
Young		0.20 ± 0.08	0.11 ± 0.04	0.09 ± 0.03	0.13 ± 0.04	0.09 ± 0.05	0.08 ± 0.06
Older		0.04 ± 0.04	*−0.02 ± 0.03	*−0.02 ± 0.02	*0.00 ± 0.03	*−0.06 ± 0.04	*−0.06 ± 0.04

Changes (Δ) in hemodynamics and forearm vascular responses to 3 min of mental stress in young (*n* = 13) and older subjects (*n* = 11). FBF is reported in mL/min and FVC is reported in au. Data are Mean ± SEM. SBP, systolic blood pressure; DBP, diastolic blood pressure; MAP, mean arterial blood pressure; HR, heart rate; FBF, forearm blood flow; FVC, forearm vascular conductance; Rec, recovery. Blood pressure values are reported in units of mmHg.

* denotes a significant difference from young subjects.

**Figure 1.**
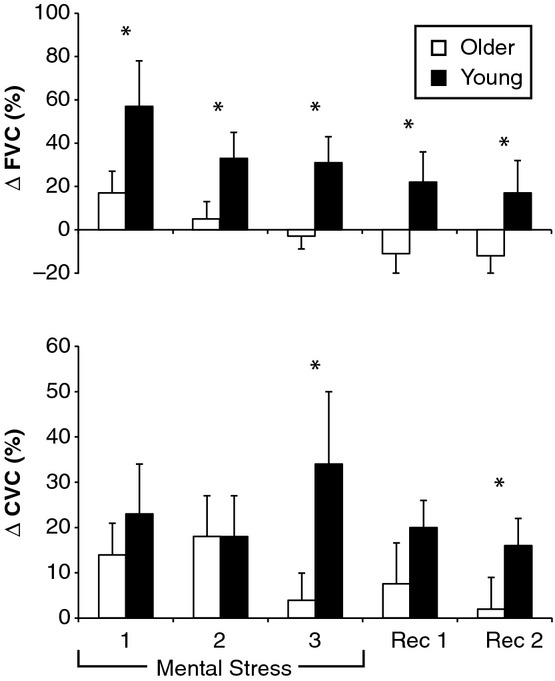
Skeletal muscle blood flow and skin blood flow responses to mental stress. Changes in forearm vascular conductance (ΔFVC, top) and cutaneous vascular conductance (ΔCVC, bottom) were determined in 13 young subjects (black bars) and 11 older subjects (white bars). Rec, recovery, Mean ± SEM, *P < 0.05 between groups at specific time point.

The ANOVA for FBF revealed a main effect for time (*P* = 0.002) and a group by time interaction (*P* = 0.048), but no main effect for group (*P* = 0.251). For FVC, there was a main effect for time (*P* = 0.020) and the treatment by time interaction nearly reached the threshold for significance (*P* = 0.059), but there was no effect of group (*P* = 0.143). As shown in Figure 1, older adults had an attenuated FVC response during the first minute (*P* = 0.044), second minute (*P* = 0.019), and third minute of mental stress (*P* = 0.017) and this effect persisted during the first (*P* = 0.033) and second (*P* = 0.043) minute of recovery. The increase in CVC was blunted in the older adults during the third minute of mental stress (*P* = 0.043) and the second minute of recovery (*P* = 0.009).

**Figure 2.**
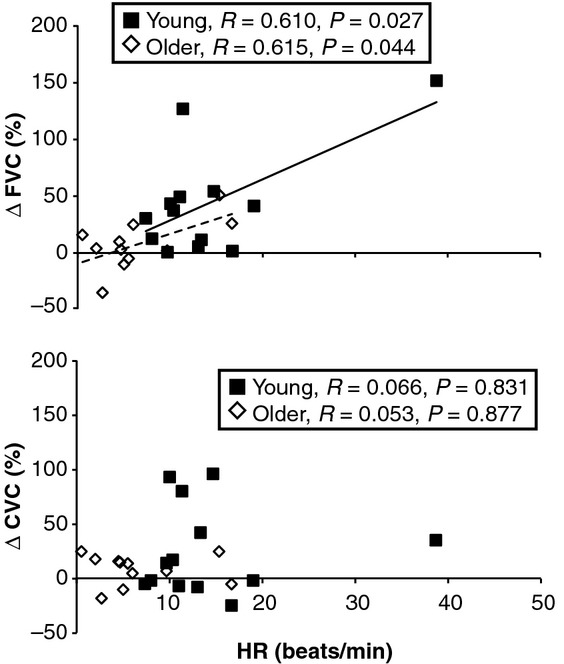
Correlations between the change in heart rate to mental stress (ΔHR) and changes in forearm vascular conductance (ΔFVC, top) and the change in cutaneous vascular conductance (ΔCVC, bottom) to mental stress. Young subjects are noted as black squares with solid line and older subjects are noted as white diamonds with a dashed line.

Figure 2 shows the relationship between ΔHR, %ΔFVC, and %ΔCVC. In young subjects, there was a significant relationship between ΔHR and %ΔFVC (*R* = 0.610, *P* = 0.027) and this relationship was also evident in older adults (*R* = 0.615, *P* = 0.044). There was no relationship between ΔHR and %ΔCVC in either group (Fig. 2, bottom).

**Figure 3.**
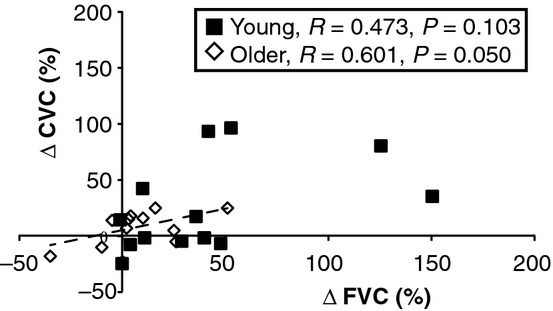
Correlation between the change in forearm vascular conductance (ΔFVC) and the change in cutaneous vascular conductance (ΔCVC) to mental stress. Young subjects are noted as black squares and older subjects are noted as white diamonds. The dashed line depicts the significant correlation in older subjects.

When evaluating the entire group of subjects, there was a significant relationship between %ΔFVC and %ΔCVC (*R* = 0.537, *P* = 0.007), but this relationship was not statistically significant when considering the young subjects alone (Fig. 3). Taken together, these correlation data (Figs. 2, 3) indicate that changes in HR due to mental stress are associated with FVC responses but not CVC response. However, FVC response and CVC responses to mental stress are related to each other in young people.

## Discussion

The purpose of this study was to compare forearm vascular responses to mental stress in young and older subjects. We hypothesized that older adults would have attenuated FVC and CVC to this acute laboratory stressor. Consistent with our hypothesis, the current data indicate that healthy older adults have less forearm vasodilation in response to mental stress compared to healthy young adults. Additionally, older adults had an attenuated increase in HR when challenged with mental stress which could partly explain the attenuated forearm vasodilator responses. These findings are important on a physiological level and may also be clinically relevant.

We found that the HR response to mental stress was blunted in older adults. Prior studies in young subjects have found that HR increases 15–35 bpm in response to laboratory mental arithmetic (Carter and Lawrence 2007; Kuipers et al. 2008; Klein et al. 2010). Our data in young subjects are consistent with these previous findings and we further the literature by reporting that older adults have an attenuated HR response to mental arithmetic such that ΔHR was approximately half that observed in young subjects (Table 2). In a previous laboratory study by Ng et al. (1994), the ΔHR was not different between groups in response to mental arithmetic. However, in this cited study (Ng et al. 1994), subjects were not verbally encouraged to respond faster as they were in our study. In a different mental stress study, Esler et al. (1995) did provide “a background of impatience and interruption” to both young and older subjects and found similar changes in HR in response to 10 min of mental arithmetic. Whether verbal encouragement or harassment influences parasympathetic withdrawal or sympathetic activation differently between age groups could be determined in future studies.

The MAP response was comparable between groups which is consistent with prior experiments from this laboratory that found similar ΔMAP in response to isometric handgrip, the cold pressor test, and voluntary apnea between healthy young and older subjects (Muller et al. 2012, 2013b; Patel et al. 2013). In another study using healthy, normotensive older adults, Ng et al. (1994) also found no group differences in response to the Stroop color word test and mental arithmetic. When considering that the older adults had smaller HR response (and probably smaller cardiac output as well), the similar MAP responses may suggest a larger rise in total peripheral resistance and/or greater blunted vasodilation in other vascular beds in the older subjects. It is also important to note that baroreflex sensitivity is impaired in older adults (Monahan 2007) and that the HR and MAP responses to other psychological stressors may show divergent responses across the lifespan (Uchino et al. 2010).

Our primary novel finding is that skeletal muscle blood flow (i.e., FBF and FVC) was blunted in older adults in response to mental stress. Indeed, FVC was not only attenuated in older adults during mental stress and recovery relative to young control subjects but was also lower than the preceding baseline (Fig. 1). These findings are consistent with our hypothesis and add to the growing body of literature that FBF responses to physiological and pharmacological interventions are impaired in people with cardiovascular risk factors (e.g., aging) (Takeshita et al. 1982; Cardillo et al. 1998; Schwartz et al. 2011). In a prior study using VOP, hypertensive subjects had an impaired FBF response to 3 min of mental arithmetic, but this effect was not observed in hypercholesterolemic subjects (Cardillo et al. 1998). A more recent study using VOP found that young prehypertensive subjects had impaired FVC to mental stress compared to young normotensive subjects (62 vs. 116%). Thus, with respect to FBF responses to mental stress, the aging process may share a common pathophysiological mechanism to hypertension.

There are two likely physiologic explanations for the differences in forearm vasodilation observed between young and older adults during mental stress. The first explanation is β‐adrenergic receptor desensitization in the elderly population (Vestal et al. 1979; Xiao and Lakatta 1992). β‐receptors play a critical role in increasing HR and contractility (mostly a β‐1 effect) as well as dilating peripheral blood vessels (mostly a β‐2 effect). Therefore, desensitized β‐receptors throughout the body could explain blunted tachycardia as well as the differences in vasodilation between the groups. The evidence for a β‐adrenergic contribution is strengthened by the relationship between ΔHR and ΔFVC in both groups (Fig. 2 top), but one must consider that changes in parasympathetic withdrawal could also be responsible for the blunted tachycardia (Machado‐Moreira et al. 2012). A second explanation could be impaired endothelial function, which is known to be impaired in elderly individuals (Eskurza et al. 2004). Because mental stress raises HR and MAP, this will enhance shear stress on the vessel wall, subsequently leading to nitric oxide production and increased FBF. However, in older adults the blunted HR may reduce the shear stimulus. Therefore, it remains unclear if the blunted FVC response to mental stress is due to impairments in central (i.e., cardiac output) and/or peripheral (i.e., local forearm vasculature) function. Additional studies using local or systemic β‐blockade may help address these issues more directly.

An additional novel finding is that skin blood flow (i.e., CVC) was blunted in older adults during the third minute of mental stress. It is clear that skin and muscle blood flow are not controlled identically (Charkoudian 2003; Holowatz et al. 2008) and the reason why skin blood flow increases during mental stress (i.e., neural vs. nonneural, direct vs. indirect) is not entirely known. Despite this lack of understanding in the literature, the current data suggest that FVC and CVC have similar temporal response patterns in response to mental stress such that a net forearm vasodilation is observed in young people, whereas older adults have impaired responses. Previous studies have also found that older adults have impaired cutaneous vasodilation in response to local heating and whole body heating (Kenney 1988; Kenney et al. 1997; Holowatz et al. 2005; Tew et al. 2011). Impairments in the nitric oxide pathway have been consistently found (Minson et al. 2002; Bruning et al. 2012), but there is less evidence for desensitized β‐adrenergic receptors in aged human skin (Crandall et al. 1997). We believe this is an important area for future study and speculate that cutaneous vasodilation in response to mental stress is differentially controlled relative to heat stress. The current skin blood flow data are not consistent with a recent study by Muller et al. (2013d), which measured CVC on the dorsal aspect of the foot in young subjects. The investigators did not observe a significant increase in CVC relative to baseline (Muller et al. 2013d). We speculate this discrepancy is due to limb differences in neural and/or vascular responsiveness to mental stress (Carter et al. 2005b; Kuipers et al. 2008; Yang et al. 2013).

It has been established that the HR response to mental stress is positively correlated to the increase in FVC such that larger tachycardia is associated with larger skeletal muscle vasodilation (Pike et al. 2009). From an evolutionary standpoint, it is perhaps not surprising that HR and FVC are related. However, subjects in this cited study (Pike et al. 2009) were aged 18–40, so it remains unclear if older adults have a similar response. Our current data (Fig. 2, dashed line) indicate that this relationship is also evident in older adults despite the narrow range of ΔHR observed in these subjects. The ΔCVC was not correlated with the ΔHR in either group which finding may be due to differential control of skin blood flow by the SNS during mental stress. It is also important to point out that in response to mental stress, larger brachial blood flow responses (proximal conduit artery) were associated with larger CVC responses (distal microcirculation) in the combined group of subjects. Brachial blood flow supplies predominantly forearm skeletal muscle. but our data suggest it may be related to forearm skin blood flow as well.

## Conclusions

In this study, we observed attenuated forearm vasodilation (i.e., both FVC and CVC) to mental stress in healthy older adults. The older adults also had an attenuated HR response to this laboratory stimulus relative to young subjects. These findings address several gaps in knowledge and may stimulate future experiments using the lower limb vasculature and/or clinical populations.

## Acknowledgments

The authors are grateful for the nursing support provided by Cheryl Blaha, Jessica Mast, and Todd Nicklas and the engineering support provided by Michael Herr. Gratitude is also extended to Anne Muller for preparing the graphics for this study and to Lawrence Sinoway and Urs Leuenberger for clinical oversight. The authors thank Amanda Ross for a critical revision of this manuscript. Finally, the authors acknowledge the administrative guidance of Kris Gray and Jen Stoner.

## Conflict of Interest

None declared.
